# Ce Filling Limit and Its Influence on Thermoelectric Performance of Fe_3_CoSb_12_-Based Skutterudite Grown by a Temperature Gradient Zone Melting Method

**DOI:** 10.3390/ma14226810

**Published:** 2021-11-11

**Authors:** Xu-Guang Li, Wei-Di Liu, Shuang-Ming Li, Dou Li, Jia-Xi Zhu, Zhen-Yu Feng, Bin Yang, Hong Zhong, Xiao-Lei Shi, Zhi-Gang Chen

**Affiliations:** 1 State Key Laboratory of Solidification Processing, Northwestern Polytechnical University, Xi’an 710072, China; leexuguang@mail.nwpu.edu.cn (X.-G.L.); lidou@mail.nwpu.edu.cn (D.L.); zhujx1107@mail.nwpu.edu.cn (J.-X.Z.); fengzhenyu97@mail.nwpu.edu.cn (Z.-Y.F.); 2016100413yb@mail.nwpu.edu.cn (B.Y.); zhonghong123@nwpu.edu.cn (H.Z.); 2 Australian Institute of Bioengineering and Nanotechnology, The University of Queensland, Brisbane, QLD 4072, Australia; weidi.liu@uq.net.au; 3 Centre for Future Materials, University of Southern Queensland, Brisbane, QLD 4300, Australia; xiaolei.shi@usq.edu.au; 4 School of Mechanical and Mining Engineering, The University of Queensland, Brisbane, QLD 4072, Australia

**Keywords:** skutterudite, CoSb_3_, Ce-filling, thermoelectric

## Abstract

CoSb_3_-based skutterudite is a promising mid-temperature thermoelectric material. However, the high lattice thermal conductivity limits its further application. Filling is one of the most effective methods to reduce the lattice thermal conductivity. In this study, we investigate the Ce filling limit and its influence on thermoelectric properties of p-type Fe_3_CoSb_12_-based skutterudites grown by a temperature gradient zone melting (TGZM) method. Crystal structure and composition characterization suggests that a maximum filling fraction of Ce reaches 0.73 in a composition of Ce_0.73_Fe_2.73_Co_1.18_Sb_12_ prepared by the TGZM method. The Ce filling reduces the carrier concentration to 1.03 × 10^20^ cm^−3^ in the Ce_1.25_Fe_3_CoSb_12_, leading to an increased Seebeck coefficient. Density functional theory (DFT) calculation indicates that the Ce-filling introduces an impurity level near the Fermi level. Moreover, the rattling effect of the Ce fillers strengthens the short-wavelength phonon scattering and reduces the lattice thermal conductivity to 0.91 W m^−1^ K^−1^. These effects induce a maximum Seebeck coefficient of 168 μV K^−1^ and a lowest *κ* of 1.52 W m^−1^ K^−^^1^ at 693 K in the Ce_1.25_Fe_3_CoSb_12_, leading to a peak *zT* value of 0.65, which is 9 times higher than that of the unfilled Fe_3_CoSb_12_.

## 1. Introduction

With the consumption of traditional fossil fuels and the aggravation of environment pollution, exploring new and effective energy utilization techniques has experienced increasing significance [1,2,3]. Thermoelectric technology, enabling the direct energy conversion between heat and electricity, has provided a promising and eco-friendly energy solution [4,5,6]. The thermoelectric performance is fundamentally characterized by the material dimensionless figure-of-merit (*zT*), defined as *zT* = *S*^2^*σT/κ*, where *S*, *σ*, *κ* and *T* are the Seebeck coefficient, electrical conductivity, thermal conductivity (comprised of electronic contribution *κ_e_* and lattice contribution *κ_l_*) and temperature in Kelvin, respectively [7,8,9]. High power factor (*S^2^σ*) and low *κ* are necessary for high *zT* [8,9]. *S*, *σ,* and *κ_e_* are related to each other as a function of other fundamental parameters, such as carrier concentration (*n*) [10,11,12]. These fundamental parameters need to be optimized. Typically, the *n* of thermoelectric materials can be optimized by valence electron counts engineering [13,14], modulation doping [15], and band gap engineering [16]. Other than the interrelated parameters, reducing *κ_l_* can achieve a low *κ* and high *zT* [17]. The reduced *κ_l_* can be achieved by introducing additional structure defects, such as point defects [18], dense grain boundaries [19], and nanoprecipitates [20,21] for strengthening phonon scattering.

Among thermoelectric materials, skutterudites, especially CoSb_3_-based skutterudites, are promising application prospect in the field of mid-temperature power generation [22,23,24]. Binary CoSb_3_ is an intermetallic compound formed by a peritectic reaction [25]. CoSb_3_ is a body-centered cubic cage-like crystal structure (*Im-3* space group) with two void positions at the 2a sites (0, 0, 0) and (1/2, 1/2, 1/2) in the unit cell [26,27]. Intrinsic CoSb_3_ is a p-type semiconductor with high *S* and high carrier mobility (*µ*) [28]. However, the strong Co-Sb covalent bonding induces high *κ_l_* (~7.5 W m^−1^ K^−1^ at room temperature) of intrinsic CoSb_3_, limiting its thermoelectric performance [29,30].

To reduce the *κ_l_* of intrinsic CoSb_3_, filling the void position at the 2a site with small atom can tune CoSb_3_ into filled-CoSb_3_ (R_x_Co_4_Sb_12_, R is the filling elements and x is the filling fraction), which possesses a feature of phonon glass and electron crystal (PGEC) [31]. The fillers are loosely bounded and rattle near the equilibrium positions and significantly scatter the low-frequency phonons, leading to decreased *κ_l_* and improved *zT* [32,33]. The fillers can be rare-earth atoms [18,34], alkaline-earth atoms [35], alkaline metals atoms [36]. Pei et al. [36] found that n-type Na_0.48_Co_4_Sb_12_ had a reduced room-temperature *κ_l_* of 3.5 W m^−1^ K^−1^ in accordance with a peak *zT* of 1.25. Alkaline-earth Ba can be filled into the void position at the 2a site of CoSb_3_ to form n-type filled-CoSb_3_ and approach a decreased *κ_l_* of 0.73 W m^−1^ K^−1^ and a peak *zT* of ~1.0 in Ba_0.51_CO_4_Sb_12_ [35]. Yb is another utilized rare-earth filler since the relatively high atomic mass and small ionic radius [18,37], which can lead to a low *κ_l_* of 0.62 W m^−1^ K^−^^1^ in n-type Yb_0.47_Co_4_Sb_12_ [38]. However, rare-earth Ce has a low filling fraction in CoSb_3_ comparing with Yb. Morelli et al. [39] prepared n-type Ce-filled CoSb_3_ by arc melting and found a low filling fraction of ~0.1. Even though with a low Ce filling fraction, the *κ_l_* was strongly depressed and a low *κ_l_* of ~4 W m^−1^ K^−1^ was obtained, which is only half of the unfilled CoSb_3_. To improve the Ce filling fraction in undoped CoSb_3_, Tang et al. [40] used phase diagram method to increase this value up to 0.2. Due to the increased filling fraction, a further reduced *κ_l_* of ~2 W m^−1^ K^−1^ and a *zT* value of 1.3 at 850 K were obtained in n-type Ce_0.14_Co_4_Sb_12_ prepared by melting-quenching-annealed-sintering. Besides, the thin film CoSb_3_ sample prepared by deposition experienced increased Ce-filling fraction. Smalley et al. [41] reported a high Ce-filling fraction of ~0.55 in deposited CoSb_3_ film.

Although heavy filling in the void position remarkably reduces the *κ_l_* of CoSb_3_-based thermoelectric materials, heavily filled CoSb_3_-based materials are generally n-type semiconductors because the fillers function as electron donors [20,34,41]. In term of the assembly of thermoelectric devices, both p-type and n-type materials are required. Hence, p-type CoSb_3_-based thermoelectric materials are necessary. To achieve low *κ_l_* of p-type CoSb_3_-based thermoelectric materials, Fe has been partially used to substitute Co [6,18,40], behaving as the electron acceptor to tune into p-type. Particularly, after Fe substitution at the Co site, CaFe_3.5_Co_0.5_Sb_12_ has a high *S*^2^*σ* of 33 μW cm^−1^ K^−2^ with a positive *S* of 170 μV K^−1^ and a low *κ_l_* of ~0.9 W m^−1^ K^−1^ at 773 K [42]. Furthermore, charge-compensational doping by the substitution at Co or Sb sites has been widely applied to increase the filling faction of Ce in bulk CoSb_3_-based skutterudites, which can simultaneously tune n-type CoSb_3_ skutterudites into p-type ones [43,44]. Tanahashi et al. [45] found a Ce filling fraction of ~0.9 in p-type CoSb_3_-based skutterudites with the nominal composition of CeFe_3_CoSb_12_, which is prepared by gas-phase atomization and sintering and approached a *zT* of 0.63 at 700 K. Chen et al. [46] reported a p-type Ce_0.95_Fe_3_CoSb_12.1_ grown by scanning laser melting method combined with spark plasma sintering and achieved the Ce filling fraction of 0.85 and the *zT* of ~0.79 at 750 K.

**Figure 1:**
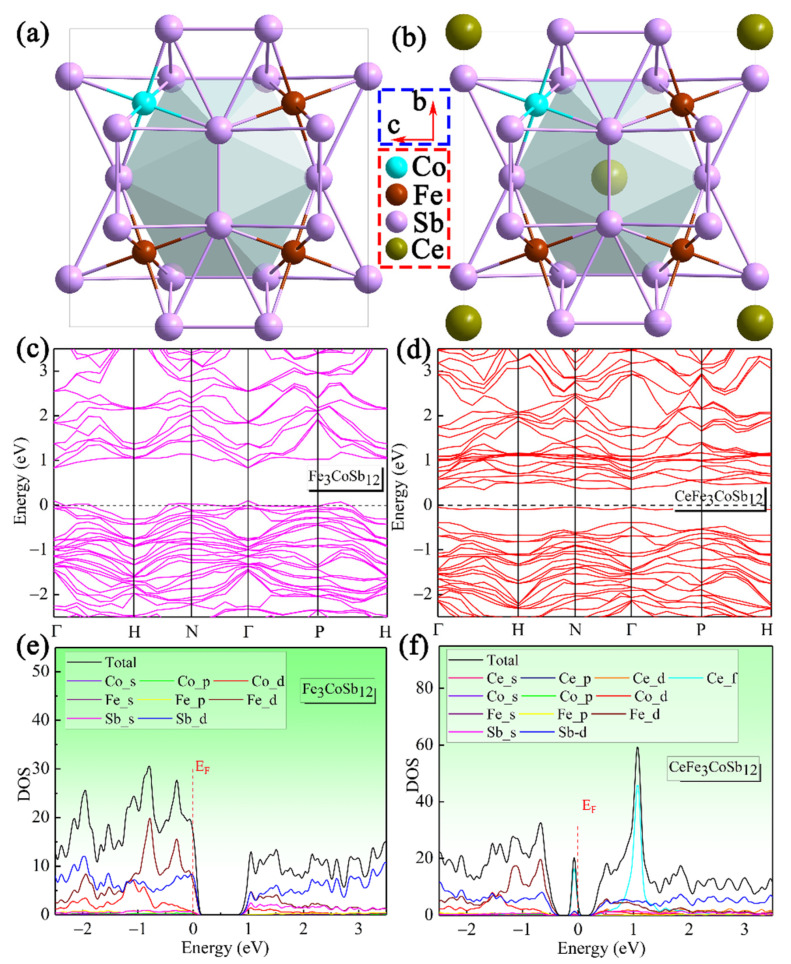
Band structures and density of states (DOS) of the unfilled and Ce-filled Fe_3_CoSb_12_-based skutterudites: The conventional unit cell of unfilled (**a**) and Ce-filled (**b**) Fe_3_CoSb_12_-based skutterudites. Calculated band structures of unfilled (**c**) and Ce-filled (**d**) Fe_3_CoSb_12_-based skutterudites and Calculated DOS of unfilled (**e**) and Ce-filled (**f**) Fe_3_CoSb_12_-based skutterudites.

As suggested in Figure 1a–f, crystal structures of the unfilled and Ce-filled Fe_3_CoSb_12_ and corresponding density functional theory (DFT)-calculated band structures and density of states (DOS) were firstly investigated. As can be seen, Ce-filling introduces an impurity level near the Fermi energy (*E_F_*), which is mainly contributed by the *f* orbital of Ce atom. Besides, Ce-filling also increases the slope of DOS near *E_F_* and correspondingly contributes to increased *S* [47,48]. Additionally, filled CoSb_3_-based thermoelectric materials can be fabricated by various methods, such as traditional melting-quenching-annealing-sintering [49], melt-spinning combined with spark plasma sintering technique (MS-SPS) [50], high-energy ball-milling combined with hot pressing (BM-HP) technique [51], and temperature gradient zone melting (TGZM) method [52,53]. Among them, TGZM is a novel material preparation method, which can synthesize CoSb_3_-based skutterudites with faster speed and higher purity by avoiding the complex peritectic solidification process. Effects of Ce fillers and its filling fraction in TGZM-prepared Fe_3_CoSb_12_ might be different from that prepared by other methods. In this study, we employed the novel TGZM method to investigate the influence and filling fraction of Ce in Fe_3_CoSb_12_. A series of Ce-filled p-type Ce_x_Fe_3_CoSb_12_ (x = 0 to 1.5) samples were prepared. We found that the maximum filling level of Ce is 0.73 in the TGZM-prepared Ce_1.25_Fe_3_CoSb_12_ with a measured composition of Ce_0.73_Fe_2.73_Co_1.18_Sb_12_. The synergistic effect on Fe substitution at the Co and Ce-filling result in reduced *n_H_* to 1.03 × 10^20^ cm^−3^ in Ce_1.25_Fe_3_CoSb_12_. A low *κ_l_* of 0.91 W m^−1^ K^−1^ at 693 K can be obtained in the Ce_1.25_Fe_3_CoSb_12_, significantly contributing to an increased *zT* of 0.65 at 693 K.

## 2. Materials and Methods

### 2.1. Samples Preparation

Initial ingots of (Fe_3_Co)-95 at. % Sb filled with x at. % Ce (x = 0, 0.25, 0.5, 0.75, 1, 1.25, 1.5) were prepared by induction melting at 1473 K for 30 min in a vacuum induction furnace (Xi’an, China) (~10^−3^Pa) followed by a furnace cooling. Elements Fe (99.95 at. %), Co (99.95 at. %), Sb (99.995 at. %), and Ce (99.9%), purchased from CNBM (Chengdu, China) Optoelectronic Materials Com., Ltd., were properly weighed as raw materials according to the nominal compositions. Extra Sb was added to obtain the samples of nominal composition Ce_x_Fe_3_CoSb_12_ after the TGZM process. Besides, extra Ce was introduced to compensate the Ce loss during TGZM process. The as-cast ingots were cut into cylinder samples with a diameter of 12.8 mm, and the oxide layer on the surface was cleaned before being put into a high-purity alumina tube with an inner diameter of 13 mm to execute the TGZM process, which was described in detail in previous reports [54,55]. The TGZM process was then conducted in a homemade directional solidification furnace of a thermal stabilization time of 48 h with an estimated temperature gradient of 40 K/mm. The TGZM-grown samples were obtained from the mushy zone formed during the TGZM process. The obtained samples were cleaned, polished, and carried out microstructure characterization and performance tests. Samples throughout the manuscript are described as their nominal compositions after the TGZM process.

### 2.2. Microstructure Characterization

The crystal structures of as-fabricated samples were determined by powder X-ray diffraction (XRD-7000, Shimadzu, Japan) with Cu-K_α_ radiation. The lattice parameters were obtained by Rietveld analysis. Scanning electron microscopy (SEM, Verios G4, FEI, equipped with EDS, Hillsboro, OR, USA) was used to acquire the morphologies of the sample’s surface and a Double Cs Corrector Transmission Electron Microscope (Cs-TEM, Themis Z, FEI, Hillsboro, OR, USA) was used to characterize their microstructure and chemical features. The actual chemical content was obtained by taking the average of fifteen different positions of each sample. Electron backscattered diffraction (EBSD, Thermo QuasOr type, Waltham, MA, USA) attached to FEI Helios G4 CX type SEM (Hillsboro, OR, USA)was used to determine the crystal orientation relationship.

### 2.3. Properties Measurements

The samples with 12.7 mm in diameter and 1.5 mm in thickness were used to measure their thermoelectric properties from 303 K to 813 K. In our measurements, *σ* and *S* were measured simultaneously using the LSR-3 system (Linseis, Zelb, Bavaria, Germany) under the helium atmosphere. *κ* was calculated by *κ = D·C_P_·ρ*, where *D* is the thermal diffusivity measured by LFA-1000 (Linseis, Zelb, Bavaria, Germany), *C_P_* the specific heat obtained by DSC (STA-449C, Netzsch, Germany), and *ρ* the density obtained by the Archimedes method. The Hall coefficient (*R*_H_), measured on the PPMS system (CFMS-14T, London, UK) with a magnetic field of ±2T, was used to calculate the room-temperature Hall carrier concentration (*n_H_*), determined by the formula [18]: *n_H_* = 1/*eR_H_*, where *e* represents the electron charge.

### 2.4. Density Functional Theory (DFT) Calculations

The physical properties of Fe_3_CoSb_12_, CeFe_3_CoSb_12_ were calculated using the Cambridge Serial Total Energy Package (CASTEP) [56]. The exchange-correlation interactions were described using the generalized gradient approximation (GGA) with the Perdew-Burke-Ernzerhof (PBE) type [57]. The plane wave cutoff energy was 450 eV for geometry optimization, band structures, and density of states calculations. The Monkhorst-Pack grid parameters were set to 8 × 8 × 8 (34 Irreducible k-points) for calculations. The Convergence tolerances were set to 1.0 × 10^−5^ eV/atom, 0.03 eV/Å, 0.05 GPa and 0.001 Å for energy, maximum force, maximum stress and maximum displacement, respectively.

## 3. Results and Discussion

### 3.1. Microstructure and Phase Composition

**Figure 2:**
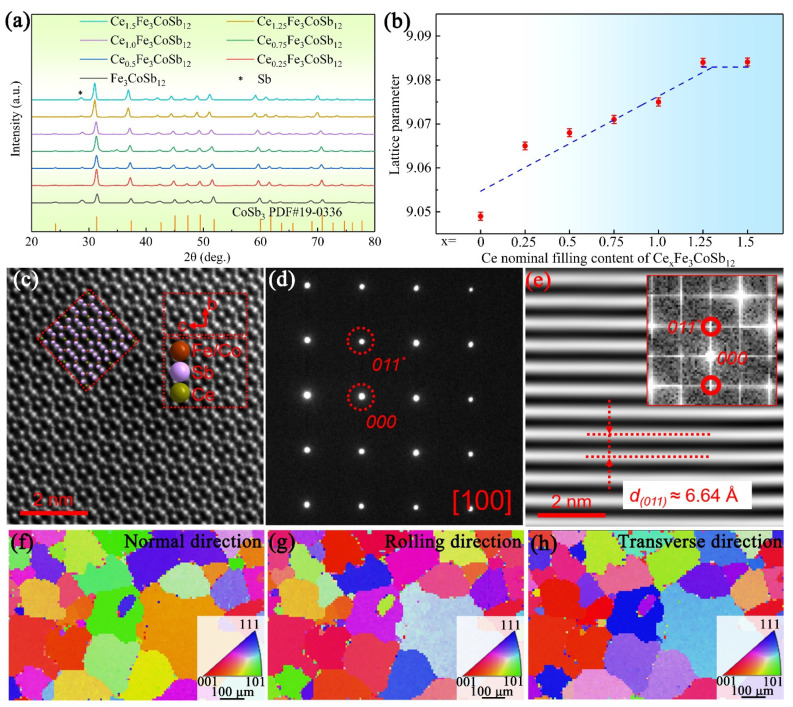
(**a**) XRD diffraction patterns and (**b**) Lattice parameter *a* of the as-prepared Ce_x_Fe_3_CoSb_12_ (x = 0 to 1.5). (**c**) HAADF image with an inserted 2 × 2 × 2 supper cell model, (**d**) SAED pattern in (**c**) and (**e**) Inverse pole figure (IPF) maps in CeFe_3_CoSb_12_. The EBSD IPF maps in (**f**) normal direction, (**g**) rolling direction and (**h**) transverse direction of the Ce_1.25_Fe_3_CoSb_12_.

To understand crystal structures of the as-prepared Ce_x_Fe_3_CoSb_12_ (x = 0 to 1.5), we firstly investigated powder X-ray diffraction (XRD) patterns and the results are shown in Figure 2a. Main diffraction peaks of samples can be identified as the body-centered cubic CoSb_3_ with a lattice parameter *a* of 9.034 Å and a space group of *Im-3* (JCPDS 19-0336). Due to the characteristic of eutectic reaction, a small number of Sb impurities are observed. Figure 2b plots the corresponding *a* of the as-prepared Ce_x_Fe_3_CoSb_12_ (x = 0 to 1.5). The calculated *a* increases with increasing the Ce-filling content and stabilizes at the x of 1.25. The increased *a* should be attributed to Ce-filling induced lattice expansion. TEM investigations were carried out to further understand the crystal structure of the as-fabricated Ce_x_Fe_3_CoSb_12_. Figure 2c is an atomic-resolution TEM high-angle annular dark-field (HAADF) image of CeFe_3_CoSb_12_. The inset of Figure 2c is superimposed a 2 × 2 × 2 supper cell model for the CeFe_3_CoSb_12_ along the [100] direction, well-matched with the observed lattice. Figure 2d is a selected area electron diffraction (SAED) pattern and can be indexed along the [100] zone-axis. Figure 2e presents the corresponding inverse Fast Fourier transform (IFFT) image along with the (011) planes. The observed *d* spacing between (011) planes is ~6.64 Å, which is larger than that of CoSb_3_ (~6.39 Å). Besides, no obvious lattice distortion can be observed from Figure 2e, indicating that the as-fabricated samples have a high crystallinity. Figure 2f–h show the EBSD inverse pole figure (IPF) maps of the Ce_1.25_Fe_3_CoSb_12_. As can be seen, no obvious texture information can be observed, indicating the isotropic thermoelectric performance of the as-fabricated Ce_x_Fe_3_CoSb_12_.

**Figure 3:**
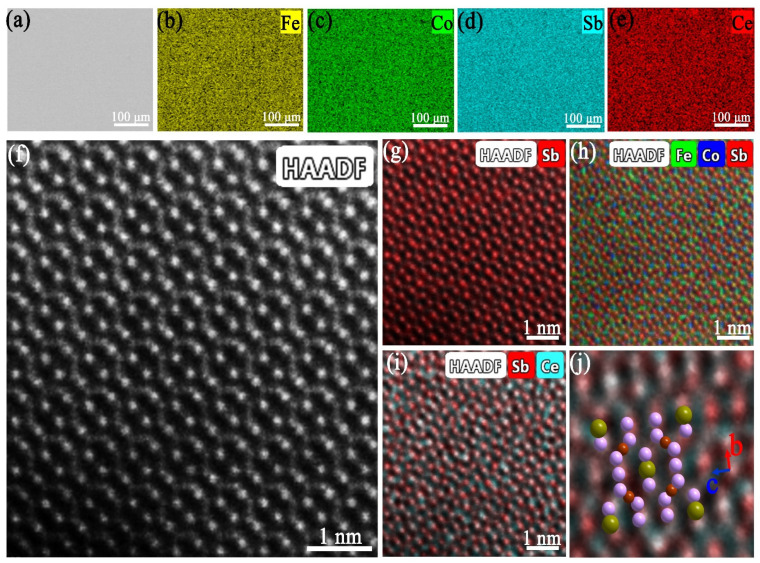
SEM image (**a**), the corresponding elemental maps of Fe (**b**), Co (**c**), Sb (**d**), Ce (**e**) of the Ce_1.25_Fe_3_CoSb_12_ sample. The HRTEM HAADF image (**f**) of the CeFe_3_CoSb_12_ and the corresponding atomic scale elemental map of Sb (**g**), overlay elemental map of Fe, Co and Sb (**h**), overlay elemental map of Sb and Ce (**i**), the magnified overlap of elemental maps superimposed with the crystal structure (**j**), where the dark green ball, the pink ball and dull-red ball represent Ce, Sb, and Fe/Co.

To understand the composition of the as-fabricated Ce_x_Fe_3_CoSb_12_, we conducted SEM image and the corresponding EDS maps of the Ce_1.25_Fe_3_CoSb_12_ and the results are shown in Figure 3a–e. As can be seen, Fe, Co, Sb, and Ce are evenly distributed in the as-fabricated Ce_1.25_Fe_3_CoSb_12_. A typical high-resolution TEM (HRTEM) HAADF image of the CeFe_3_CoSb_12_ is shown in Figure 3f. Figure 3g–i are the corresponding elemental maps in atomic scale. Figure 3j is a magnified overlap of elemental maps where the dark green balls represent Ce fillers. As can be seen, Ce filler sits at the 2a sites in the cage among the Sb-icosahedron (pink ball).

**Figure 4:**
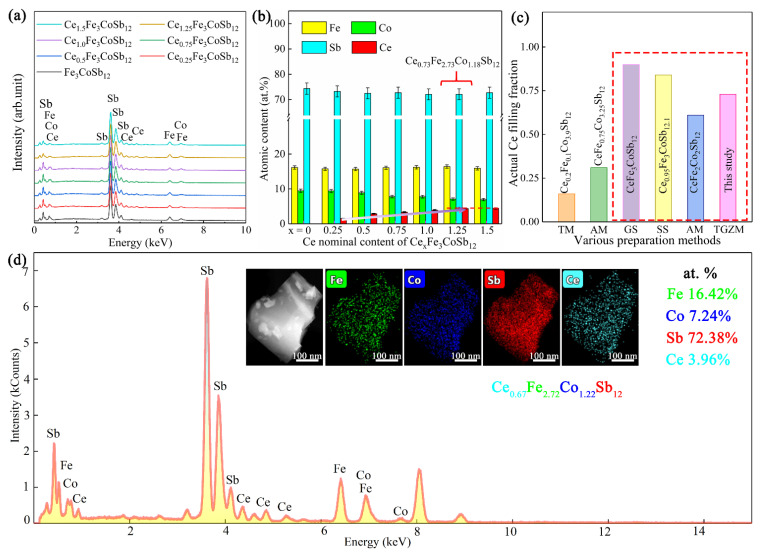
Detailed component information acquired by EDS: (**a**) EDS spectra and corresponding (**b**) average atomic ratios of Fe, Co, Sb, Ce elements in Ce_x_Fe_3_CoSb_12_ (x = 0 to 1.5) samples. (**c**) comparison of the Ce filling fraction in Fe-doped CoSb_3_ prepared by different methods, including traditional melting (TM) for Ce_0.2_Fe_0.1_Co_3.9_Sb_12_, [58] arc melting (AM) for CeFe_2_Co_2_Sb_12_ [59] and CeFe_0.75_Co_3.25_Sb_12_ [39], gas-atomized powder sintering (GS) for CeFe_3_CoSb_12_, [45] scanning laser melting and spark plasma sintering (SS) for Ce_0.95_Fe_3_CoSb_12.1_ [46] and temperature gradient zone melting (TGZM) for this study. (**d**) Typical TEM-EDS spectrum of the CeFe_3_CoSb_12_. The insert of (**d**) is the HAADF image, corresponding elemental maps and component analysis.

Figure 4a shows typical EDS spectra of the Ce_x_Fe_3_CoSb_12_. All samples are composed of Ce, Fe, Co, and Sb without other impurity elements. Figure 4b shows the average atomic ratios of Fe, Co, Sb, and Ce elements in each sample based on the statistic EDS results. As can be seen, the Ce-filling level in the samples increases at first and then tends to be stabilized at the x of 1.25, which is consistent with the peak shift from XRD. Figure 4c compares the Ce-filling fraction of Fe-doped CoSb_3_ prepared by different methods. The Ce filling fraction varies with the preparation method and Fe/Co ratio. The Fe/Co ratio in the actual composition of the samples shown in the red dotted box in Figure 4c is close to ~2.5. Under the similar Fe/Co ratio, a relative high filling fraction of 0.73 is achieved in a composition of Ce_0.73_Fe_2.73_Co_1.18_Sb_12_ prepared by the TGZM method in this study. Figure 4d shows the TEM-EDS spectrum and maps of the CeFe_3_CoSb_12_. As can be seen, Fe, Co, Sb, and Ce elements are homogeneously distributed on a micro-scale, indicating successful Fe substitution at the Co site and Ce-filling.

### 3.2. Thermoelectric Transport Properties

**Figure 5:**
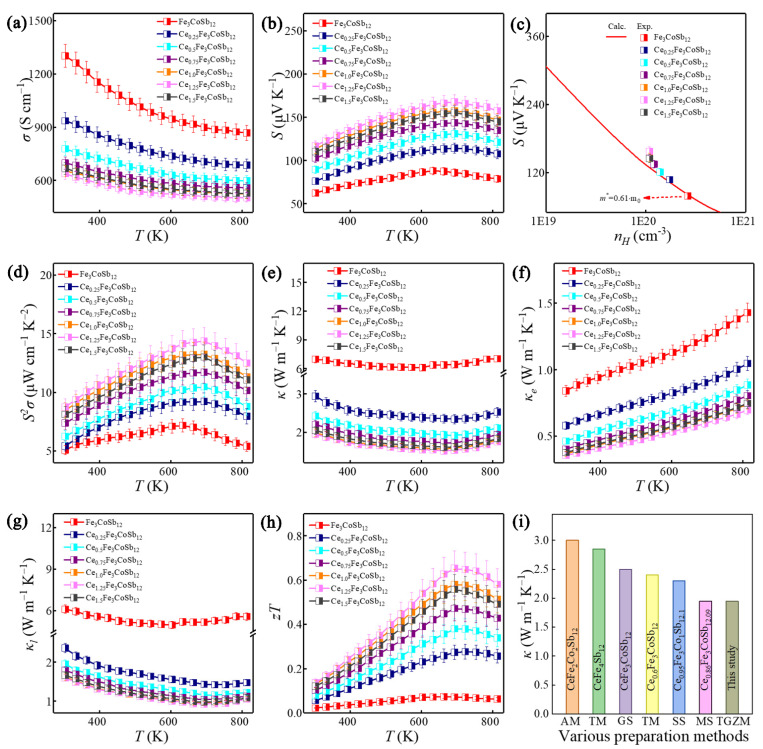
Thermoelectric properties of Ce_x_Fe_3_CoSb_12_ (x = 0 to 1.5). Temperature-dependent (**a**) *σ*; (**b**) *S*. (**c**) The experimental *S* values corresponding to the *n_H_*, where the red line represents the Pisarenko plot for Fe_3_CoSb_12_. Temperature-dependent (**d**) *S^2^σ*; (**e**) *κ*; (**f**) *κ_e_*; (**g**) *κ_l_*; (**h**) *zT*. (**i**) Comparison of the room-temperature *κ* for nominal Ce_1.25_Fe_3_CoSb_12_ with the reported values for CeFe_2_Co_2_Sb_12_ prepared by AM, [59] CeFe_4_Sb_12_ [61] and Ce_0.6_Fe_3_CoSb_12_ [62] prepared by TM, CeFe_3_CoSb_12_ prepared by GS, [45] Ce_0.95_Fe_3_CoSb_12.1_ prepared by SS, [46] and Ce_0.86_Fe_3_CoSb_12.09_ prepared by melt spinning and spark plasma sintering (MS) [63].

To understand the thermoelectric properties of the as-fabricated Ce_x_Fe_3_CoSb_12_ (x = 0 to 1.5), *σ*, *S*, *S^2^σ,* and *κ* were measured at the temperature range between 303 and 813 K. Figure 5a depicts temperature-dependent *σ* of the Ce_x_Fe_3_CoSb_12_. The Fe_3_CoSb_12_ has a higher *σ* (1302 S cm^−1^) comparing with the Ce-filled samples in the entire measured temperature. With increasing the Ce-filling level, the *σ* of the Ce_x_Fe_3_CoSb_12_ decreases from 1302 S cm^−1^ of the Fe_3_CoSb_12_ to 638 S cm^−1^ of the Ce_1.25_Fe_3_CoSb_12_ at 303 K. The slight increase of *σ* from x = 1.25 to 1.5 might be attributed to the slightly increased Sb content (as evidenced by the XRD peak intensity in Figure 2a). Besides, the nearly linear decrease of *σ* with increasing the temperature indicates that the as-fabricated Ce_x_Fe_3_CoSb_12_ is a degenerated semiconductor. With increasing the Ce content, *S* increases from 62 μV K^−1^ of the Fe_3_CoSb_12_ to 117 μV K^−1^ of the Ce_1.25_Fe_3_CoSb_12_ at 303 K (Figure 5b). The maximum *S* value of 168 μV K^−1^ at 693 K can be obtained in the Ce_1.25_Fe_3_CoSb_12_. The positive *S* indicates that the Ce_x_Fe_3_CoSb_12_ is p-type, which is consistent with the calculation of the band structures. A classic single parabolic band (SPB) model was used to evaluate the effective mass m* as described by Equations (1)–(4) [18,60]:

(1) $$S=\pm \frac{k_{B}}{e}\left(\frac{2F_{1}\left(\eta \right)}{F_{0}\left(\eta \right)}-\eta \right)$$

(2) $$m^{*}=\frac{h^{2}}{2k_{B}T}\left[\frac{nr_{H}}{4\pi F_{1/2}\left(\eta \right)}\right]$$

(3) $$r_{H}=\frac{3}{2}\frac{F_{1/2}\left(\eta \right)F_{-1/2}\left(\eta \right)}{2F_{0}^{2}\left(\eta \right)}$$

(4) $$F_{n}\left(\eta \right)=\int_{0}^{\infty }\frac{x^{n}}{1+e^{x-\eta }}dx$$

where *e* is the electron charge, *η* the reduced Fermi energy, *η* = *E_F_*/*k_B_T*, *F_n_*(*η*) the Fermi integral and *r_H_* the Hall factor. Figure 5c shows the Pisarenko plot of Fe_3_CoSb_12_ with the measured room-temperature *n_H_* and corresponding *S* of Ce-filled Fe_3_CoSb_12_ samples. With increasing Ce-filling level, the experimental *S* values corresponding to the *n_H_* deviates from the Pisarenko plot to a higher level, indicating that Ce-filling led to an increase of the m*. This should be attributed to the increased DOS near the edge of the band structure (Figure 1e,f) induced by Ce-filling. The maximum *S^2^σ* (Figure 5d) significantly increases from 6.7 μW cm^−1^ K^−2^ of the Fe_3_CoSb_12_ to 14.4 μW cm^−1^ K^−2^ of the Ce_1.25_Fe_3_CoSb_12_ at 693 K.

Figure 5e plots temperature-dependent *κ* of the as-fabricated Ce_x_Fe_3_CoSb_12_ (x = 0 to 1.5). With increasing the Ce-filling level, the *κ* gradually decreases and approaches 1.52 W m^−1^ K^−1^ of Ce_1.25_Fe_3_CoSb_12_ at 693 K. The *κ_e_* can be calculated as *κ_e_* = *LσT* (Figure 5f), where *L* is the Lorenz number calculated based on SPB model. With increasing the Ce-filling level, the *κ_e_* significantly reduces due to reduced *σ*. Figure 5g presents temperature-dependent *κ_l_*, calculated by *κ-κ_e_*. The *κ_l_* of the Ce-filled Fe_3_CoSb_12_ is much lower than that of the unfilled Fe_3_CoSb_12_. The *k_l_* reduces with increasing the Ce-filling level and approaches a lowest *κ_l_* of 0.91 W m^−1^ K^−1^ at 693 K in the Ce_1.25_Fe_3_CoSb_12_. This should be primarily attributed to the rattling effect for strengthening short-wavelength phonon scattering, induced by Ce-filling [64]. Figure 5h displays temperature-dependent *zT* of the as-fabricated Ce_x_Fe_3_CoSb_12_ (x = 0 to 1.5). Benefitting from the enhanced *S^2^σ* and significantly reduced *k*, a peak *zT* value of 0.65 can be achieved in the Ce_1.25_Fe_3_CoSb_12_ at 693 K, which is 9 times higher than that of the unfilled Fe_3_CoSb_12_. Figure 5i compares the room-temperature *κ* of nominal Ce_1.25_Fe_3_CoSb_12_ in this study prepared by TGZM with the reported *κ* of p-type Ce-filled and Fe-doped CoSb_3_ prepared by other methods. As can be seen, a relatively lower *κ* is obtained in TGZM prepared Ce_1.25_Fe_3_CoSb_12_, which is due to the higher Ce filling fraction with an optimized Fe/Co ratio. Besides, the maximum *zT* values of different Ce-filled and Fe-doped CoSb_3_ prepared by various methods are compared and shown in Figure S1 of the Supplementary Material, indicating a higher *zT* value can be obtained by optimizing Ce-filling fraction and Fe/Co ratio.

## 4. Conclusions

In this study, under the guidance of the DFT calculation, where Ce-filling can introduce an impurity level near the *E_F_* and increase the thermoelectric performance of the Fe_3_CoSb_12_, we have designed and prepared the p-type Ce_x_Fe_3_CoSb_12_ (x = 0 to 1.5) by a facile TGZM method. The Ce-filling limit in TGZM-prepared Fe_3_CoSb_12_ was found to be 0.73 with a measured composition of Ce_0.73_Fe_2.73_Co_1.18_Sb_12_. The filling limit is approached at the nominal composition of Ce_1.25_Fe_3_CoSb_12_. Under the synergistic effect, Fe substitution at the Co site and Ce-filling, an optimal *n_H_* of 1.03 × 10^20^ cm^−^^3^ is approached. The high *S* of 168 μV K^−1^ at 693 K due to the increase of the Ce filling level induces a high *S^2^σ* of 14.4 μW cm^−1^ K^−2^ at 693 K in the Ce_1.25_Fe_3_CoSb_12_, which is increased by 100% comparing with that of the Fe_3_CoSb_12_. The rattling effect of Ce fillers strongly strengthens phonon scattering, leading to reduced *κ_l_* as low as 0.91 W m^−1^ K^−1^ at 693 K in the Ce_1.25_Fe_3_CoSb_12_. Benefiting from the low *κ* of 1.52 W m^−1^ K^−^^1^ induced by both optimized *n_H_* and reduced *κ_l_*, a peak *zT* value of 0.65 at 693 K can be achieved in the Ce_1.25_Fe_3_CoSb_12_, which is 9 times higher than the Fe_3_CoSb_12_.

## Data Availability

The data presented in this study are available upon request from the corresponding author.

## Supplementary Materials

The following are available online at https://www.mdpi.com/article/10.3390/ma14226810/s1, Comparison of the maximum *zT* value of nominal Ce_1.25_Fe_3_CoSb_12_ in this study prepared by TGZM with the reported *zT* of p-type Ce-filled and Fe-doped CoSb_3_ prepared by other methods.
