# Supplementary material for: Ce Filling Limit and Its Influence on Thermoelectric Performance of Fe3CoSb12-Based Skutterudite Grown by a Temperature Gradient Zone Melting Method
Source: Materials (Basel). 2021 Nov 11;14(22):6810. doi: 10.3390/ma14226810 (PMC8620759; doi:10.3390/ma14226810)
Supplement: Supplementary file 1 [file materials-14-06810-s001.zip › materials-1415071-supplementary.pdf]

materials MDPI

Supporting Information

Ce Filling Limit and Its Influence on Thermoelectric Performance of Fe_3CoSb_{12}-Based Skutterudite Grown by a Temperature Gradient Zone Melting Method

Xu-Guang Li [1,†], Wei-Di Liu [2,3,†], Shuang-Ming Li [1,*], Dou Li [1], Jia-Xi Zhu [1], Zhen-Yu Feng [1], Bin Yang [1], Hong Zhong [1], Xiao-Lei Shi [3,4] and Zhi-Gang Chen [3,4,*]

[1] State Key Laboratory of Solidification Processing, Northwestern Polytechnical University, Xi'an 710072, China; leexuguang@mail.nwpu.edu.cn (X.-G.L.); lidou@mail.nwpu.edu.cn (D.L.); zhujx1107@mail.nwpu.edu.cn (J.-X.Z.); fengzhenyu97@mail.nwpu.edu.cn (Z.-Y.F.); 2016100413yb@mail.nwpu.edu.cn (B.Y.); zhonghong123@nwpu.edu.cn (H.Z.)
[2] Australian Institute of Bioengineering and Nanotechnology, The University of Queensland, Brisbane, QLD 4072, Australia; weidi.liu@uq.net.au
[3] Centre for Future Materials, University of Southern Queensland, Brisbane, QLD 4300, Australia; xiaolei.shi@usq.edu.au
[4] School of Mechanical and Mining Engineering, The University of Queensland, Brisbane, QLD 4072, Australia
* Correspondence: lsm@nwpu.edu.cn (S.-M.L.); zhigang.chen@usq.edu.au (Z.-G.C.)
† These authors contributed equally to this work.

Keywords: Skutterudite; $CoSb_3$; Ce-filling; Thermoelectric

1. Comparison of the maximum zT value

Figure S1 compares the maximum zT values of nominal $Ce_{1.25}Fe_3CoSb_{12}$ in this study prepared by TGZM with the reported zT of p-type Ce-filled and Fe-doped $CoSb_3$ prepared by other methods. As can be seen, our obtained zT value is comparable to $Ce_{0.6}Fe_3CoSb_{12}$ and $Ce_{0.6}Fe_3CoSb_{12}$ prepared by traditional melting (TM) method. Besides, a higher zT value can be obtained by optimizing Ce-filling fraction and Fe/Co ratio.

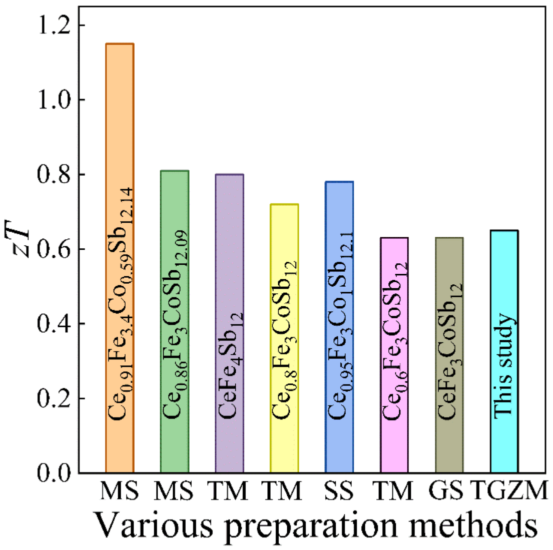

Figure S1. Comparison of the maximum zT value for nominal $Ce_{1.25}Fe_3CoSb_{12}$ with the reported values for $Ce_{0.91}Fe_{3.4}Co_{0.59}Sb_{12.14}$ [1] and $Ce_{0.86}Fe_3CoSb_{12.09}$ [2] prepared by melt spinning and spark plasma sintering (MS), $CeFe_4Sb_{12}$,[3] $Ce_{0.6}Fe_3CoSb_{12}$ and $Ce_{0.8}Fe_3CoSb_{12}$ [4] prepared by traditional

melting (TM), $CeFe_3CoSb_{12}$ [5] prepared by gas-atomized powder sintering (GS), $Ce_{0.95}Fe_3CoSb_{12.1}$ [6] prepared by scanning laser melting and spark plasma sintering SS.

References

1. Lee, S.; Lee, K.H.; Kim, Y.M.; Kim, H.S.; Snyder, G.J.; Baik, S.; Kim, S.W. Simple and efficient synthesis of nanograin structured single phase filled skutterudite for high thermoelectric performance. *Acta Mater.* **2018**, *142*, 8–17.
2. Bae, S.H.; Lee, K.H.; Choi, S.-M. Effective role of filling fraction control in p-type CexFe3CoSb12 skutterudite thermoelectric materials. *Intermetallics* **2019**, *105*, 44–47.
3. Tan, G.; Liu, W.; Wang, S.; Yan, Y.; Li, H.; Tang, X.; Uher, C. Rapid preparation of $CeFe_4Sb_{12}$ skutterudite by melt spinning: Rich nanostructures and high thermoelectric performance. *J. Mater. Chem. A* **2013**, *1*, 12657–12668.
4. Tan, G.J.; Wang, S.Y.; Tang, X.F. Thermoelectric Performance Optimization in p-Type $Ce_yFe_3CoSb_{12}$ Skutterudites. *J. Electron. Mater.* **2014**, *43*, 1712–1717.
5. Tanahashi, H.; Ohta, Y.; Uchida, H.; Itsumi, Y.; Kasama, A.; Matsubara, K. Formation of cerium-filled skutterudite thermoelectric materials in the sintering of gas-atomized powder. *J. Jpn. Inst. Metals* **2001**, *65*, 955–960.
6. Chen, F.; Liu, R.; Yao, Z.; Xing, Y.; Bai, S.; Chen, L. Scanning laser melting for rapid and massive fabrication of filled skutterudites with high thermoelectric performance. *J. Mater. Chem. A* **2018**, *6*, 6772–6779.
